# Prognostic impact of peripheral natural killer cells in primary central nervous system lymphoma

**DOI:** 10.3389/fimmu.2023.1191033

**Published:** 2023-06-22

**Authors:** Zhiguang Lin, Jingjing Ma, Yan Ma, Qing Li, Hui Kang, Mengxue Zhang, Bobin Chen, Rong Xia

**Affiliations:** ^1^ Department of Hematology, Huashan Hospital, Fudan University, Shanghai, China; ^2^ Department of Blood Transfusion, Huashan Hospital, Fudan University, Shanghai, China

**Keywords:** primary central nervous system lymphoma, B-cell lymphoma, natural killer cells, prognosis, tumor microenvironment

## Abstract

**Background:**

Primary central nervous system lymphoma (PCNSL) is an aggressive extranodal non-Hodgkin lymphoma with a poor prognosis. We aimed to evaluate the prognostic impact of circulating NK cells in PCNSL.

**Materials and methods:**

Patients diagnosed with PCNSL who were treated at our institution between December 2018 and December 2019 were retrospectively screened. Patient variables including age, sex, Karnofsky performance status, diagnostic methods, location of lesions, lactate dehydrogenase, cerebrospinal fluids (CSF), and vitreous fluids involvement or not were documented. NK cell count and NK cell proportion (NK cell count/lymphocyte count) in the peripheral blood were evaluated by flow cytometry. Some patients underwent two consecutive NK cell tests before and three weeks after chemotherapy (before the next chemotherapy). The fold change in NK cell proportion and NK cell counts were calculated. CD56-positive NK cells in tumor tissue were assessed by immunohistochemistry. NK cell cytotoxicity assay was performed using flow cytometry.

**Results:**

A total of 161 patients with PCNSL were included in this study. The median NK cell count of all NK cell tests was 197.73/μL (range 13.11–1889.90 cells/μL). The median proportion of NK cells was 14.11% (range 1.68–45.15%) for all. Responders had a higher median NK cell count (*p*<0.0001) and NK cell proportion (*p*<0.0001) than non-responders. Furthermore, Responders had a higher median fold change in NK cell proportion than non-responders (*p*=0.019) or patients in complete remission/partial remission (*p*<0.0001). A higher median fold change in NK cell count was observed in responders than in non-responders (*p*=0.0224) or patients in complete remission/partial remission (*p*=0.0002). For newly diagnosed PCNSL, patients with a high NK cell count (>165 cells/μL) appeared to have a longer median overall survival than those with a low NK cell count (*p*=0.0054). A high fold change in the proportion of NK cells (>0.1957; *p*=0.0367) or NK cell count (>0.1045; *p*=0.0356) was associated with longer progression-free survival. Circulating NK cells from newly-diagnosed PCNSL demonstrated an impaired cytotoxicity capacity compared to those from patients with PCNSL in complete remission or healthy donors.

**Conclusion:**

Our study indicated that circulating NK cells had some impact on the outcome of PCNSL.

## Introduction

Primary central nervous system lymphoma (PCNSL) is a rare subtype of lymphoma that affects the brain, eyes, spinal cord, and leptomeninges. It represents 4% of central nervous system neoplasms and 4–6% of extranodal non-Hodgkin lymphomas in immunocompetent patients (1). The mean age at diagnosis is about 63 years, and patients older than 70 years have the highest incidence. The most frequent histological subtype of PCNSL is diffuse large B cell lymphoma (DLBCL), diagnosed in up to 84.6% of patients, followed by indolent B cell lymphoma (3.2%). About 9% of patients present an unconfirmed histopathological subtype (2). PCNSL is an incurable disease with a 5-year overall survival (OS) of 26%–44.8% (3, 4). Several prognostic factors were identified to stratify the risk groups of PCNSL. Age, Eastern Cooperative Oncology Group performance status (ECOG-PS), lactate dehydrogenase (LDH), cerebrospinal fluid (CSF) protein concentration, deep brain involvement, and multifocal lesions have been proposed as parameters in various prognostic scoring systems (5).

The composition of the tumor microenvironment (TME) has emerged as a predictor of the outcome of PCNSL. CD4+ T cells were found to be correlated with longer OS or disease-free survival in PCNSL (6). Higher expression of PD-L1 in immune cells has been associated with longer OS (7). M2 macrophages, which had been shown to play an immunosuppressive role and promote tumor cell survival in many types of tumors, have been correlated with poor outcomes in PCNSL (8). Next-generation sequencing analysis has indicated that genes associated with the cytoskeleton, cell adhesion, the extracellular matrix, and matrix metalloprotease were predictors of prognosis (9). Transcriptomics analysis has revealed three immune subtypes in PCNLS: immune rich, poor, and intermediate; patients in the immune rich group had a hyperactivation of STAT3 signaling and inflammatory signaling such as interferon-γ and tumor necrosis factor-α and had a longer progression-free survival (PFS) than those in the immune intermediate or immune poor group (10).

Natural killer (NK) cells are cytotoxic innate immune cells that play a pivotal role in eliminating transformed cells and intracellular pathogens. Two main subsets of NK cells can be found in the peripheral blood: CD56dim CD16+ NK cells and CD56bright CD16- NK cells. Most circulating NK cells belong to the subset CD56dim that harbors the capacity for cytotoxicity. The CD56bright subset resides in secondary lymphoid organs or tissues and exhibits immune modulatory functions by secreting cytokines and shapes the adaptive immune response by interacting with antigen-presenting cells such as dendritic cells and macrophages (11, 12). Within the TME, NK cell infiltration has been correlated with a favorable prognosis (13–15). However, NK cells in the TME exhibit a divergent state. Some NK cells appear to have a potent cytolytic capacity, while others demonstrate dysfunctional phenotypes (16). Factors within the TME such as hypoxia, transforming growth factor-β, prostaglandin-E2, and various cell types including fibroblasts, stromal cells, regulatory T cells, and myeloid-derived suppressor cells impair NK cell function by down-regulating activating receptors or decreasing NK-cell cytotoxicity and survival (17). Regarding circulating NK cells, previous studies have indicated that a decrease in peripheral NK cell counts could be found in colorectal cancers compared to healthy controls (18); and a lower circulating NK cell count has been identified as a predictor of shorter OS in gastric cancer (19). Patients with a lower NK cell count have also been reported to have a shorter OS in chronic lymphocytic leukemia (20) and mantle cell lymphoma (21).

However, despite being an important part of the TME, little is known about the role of NK cells in PCNSL. NK cell lymphopenia in the peripheral blood has been reported to occur in up to 40% of patients with PCNSL (22). In this retrospective cohort study, our objective was to investigate the prognostic value of peripheral NK cells in PCNSL.

## Patients and methods

### Patient selection

Patients diagnosed with PCNSL who were treated at our institution between December 2018 and December 2019 were selected for the study. Patients, previously treated or untreated, aged 18 years or older with a histologically or cytologically confirmed diagnosis of PCNSL were included. Those who had received checkpoint inhibitors before or with concomitant autoimmune disease were excluded. All patients had a measurable disease status on contrast-enhanced magnetic resonance imaging. The medical records of each patient were checked by two experts. All patients were followed up until 1 January 2022 or death. The study involving human participants was reviewed and approved by the Ethics Committee of Huashan Hospital, Fudan University (ChiCTR2200055215). The patients provided their written informed consent to participate in this study.

### Data collection

Patient variables including age, sex, Karnofsky performance status (KPS), diagnostic methods (surgical excision or nonsurgical excision), number of lesions (solitary or multifocal), location of lesions, LDH, CSF involvement or not, and vitreous fluid involvement or not were documented. NK cell count and NK cell proportion (NK cell count/lymphocyte count) in the peripheral blood were evaluated by flow cytometry. Briefly, whole blood (50 μL) was stained with BD Multitest 6-Color TBNK Reagent (BD Biosciences). NK cells, which were defined as CD3^-^CD56^+^ and/or CD16^+^ cells, were measured by flow cytometry (BriCyte E6; Mindray).

Some patients underwent two consecutive NK cell tests before and three weeks after chemotherapy (before the next chemotherapy). The fold change in the proportion of NK cells was defined as (NK cell proportion_second_-NK cell proportion_first_)/NK cell proportion_first_. The fold change in NK cell count was defined as (NK cell count_second_-NK cell count_first_)/NK cell count_first_. The state of the disease of a patient at the time of NK cell analysis was documented according to the criteria of the International PCNSL Collaborative Group (IPCG) (23). Patients who were in complete remission (CR) or partial remission (PR) were regarded as responders, while those who were in stable disease (SD) or progressive disease (PD) were categorized as nonresponders. When analyzing the fold change in NK cell count/proportion, responders were defined as the patients who reached PR/CR from PD/SD or patients with newly-diagnosed PCNSL reaching PR/CR after chemotherapy; nonresponders were defined as the patients remaining in PD/SD after chemotherapy.

In the first part of this study, a cross-sectional study was conducted to analyze the correlation between NK cell-associated parameters and disease state. Newly-diagnosed PCNSL and previously treated PCNSL were put together for analysis. In the second part of this study, a retrospective cohort study was conducted to analyze NK cell-associated parameters’ impact on the prognosis of PCNSL. Only newly-diagnosed PCNSL were included in this part. PFS was defined as the time from the first NK cell analysis to disease progression, relapse, death, or censored alive. OS was defined as the time from the first NK cell analysis to death or censored alive.

### Immunohistochemical assessment of CD56-positive NK cells in tumor tissue

CD56-positive NK cells in tumor tissue were assessed by immunohistochemistry. Formalin-fixed paraffin-embedded tumor tissues were used for immunohistochemical staining with the monoclonal antibody CD56 as previously described (24). Staining was performed on the Dako Autostainer Link 48 platform (primary antibody for CD56: Clone 56C04, Xiamen Talent Biomedical Technology, China). CD56 expression was considered positive if more than 5% of immune cells were stained.

### *In vitro* cytotoxicity assay

Peripheral blood mononuclear cells (PBMC) were extracted from three healthy donors, three patients with newly-diagnosed PCNSL, and three patients with PCNSL in CR. The NK cell percentage of PBMC was determined by BD Multitest 6-Color TBNK Reagent (BD Biosciences). Cytotoxicity assay was performed as previously reported (25). An appropriate volume of PBMC was added to 0.1ml DiO-labeled K562 (Beyotime, China) so that fixed numbers of NK cells were tested in the assay. The cell mixtures were kept at 37°C in a CO_2_ incubator for 4 hours. After incubation, the cell mixtures were washed with PBS and labeled with TruStain FcX™ (Fc Receptor Blocking Solution), PE/Cyanine 7 anti-CD3 (UCHT1), PE anti-CD56 (NCAM), and APC/Cyanine 7 anti-CD45 (HI30) (Biolegend, USA) according to the manufacturer’s instructions. The percentage of dead cells within DiO-positive K562 cells was measured by flow cytometry after staining with 7-AAD.

### Statistical analysis

The Mann-Whitney test or the Kruskal-Wallis test was used to compare different groups of NK cell proportion/count. PFS and OS were estimated using the Kaplan-Meier method. The difference in PFS and OS between the groups was analyzed using a logarithmic rank test. The Cox regression model was used for the multivariate analysis, and factors with a *p*-value less than 0.1 were included in the multivariate analysis. Cut-off values of NK cell proportion/count were calculated by receiver operating characteristic (ROC) curves. A *p*-value less than 0.05 was considered statistical significance. All statistical analyzes were performed using the GraphPad Prism 9.0 software package and the STATA 15 software package.

## Results

### Characteristics of patients undergoing NK cell analysis

A total of 161 patients with PCNSL from December 2018 to December 2019 at our institution were included in this retrospective study. Eighty-nine patients (55.28%) were newly diagnosed PCNSL and the remaining 72 (44.72%) were previously treated. The disease characteristics are detailed in **Table 1**. The median age of the patients in the cohort was 60 years (range 24–82). Thirty-four patients (21.12%) underwent one peripheral blood NK cell analysis and the remaining 127 (78.88%) patients underwent two consecutive peripheral blood NK cell analyses before and after chemotherapy. Among the 288 documented NK cell analyses, 156 (57.14%) were performed when PD was documented. The median NK cell count of all NK cell tests was 197.73/μL (range 13.11–1889.90 cells/μL). The median proportion of NK cells (NK cells/lymphocytes) was 14.11% (range 1.68–45.15%) for all.

**Table 1:** Clinical characteristics of patients.

Characteristics	All patients (n=161)
Age (years)	
Median (range) ≥60 y <60 y	60 (24–82) 83 (51.55%) 78 (48.45%)
Sex	
Male Female	79 (49.07%) 82 (50.93%)
Newly diagnosed PCNSL	
Yes No	89 (55.28%) 72 (44.72%)
Number of NK cell tests	
One Two	34 (21.12%) 127 (78.88%)
Disease state	Number of NK cell analyses (n=288)
Complete remission Partial remission Stable disease Progressive disease	58 (20.14%) 58 (20.14%) 16 (5.55%) 156 (54.17%)
NK cell count (cells/μL)	
Median (range) Responders Non-responders	197.73 (13.11–1889.90) 246.12 (44.23–1306.30) 162.47 (13.1–1889.90)
Disease state	
Median (range) Responders Non-responders	14.11 (1.68–45.15) 17.44 (4.48–45.02) 11.42 (1.68–45.15)
Fold change of NK cell proportion	
Median (range) Responders Non-responders CR/PR	0.20 (-0.55–8.88) 0.75 (-0.55–8.88) 0.13 (-0.38–5.05) -0.05 (-0.44–0.43)
Fold change of NK cell count	
Median (range) Responders Non-responders CR/PR	0.22 (-0.99–10.98) 0.59 (-0.70–10.70) 0.07 (-0.59–10.98) 0.01 (-0.99–2.12)

PCNSL, primary central nervous system lymphoma; CR/PR, complete remission/partial remission.

**Figure 1:**
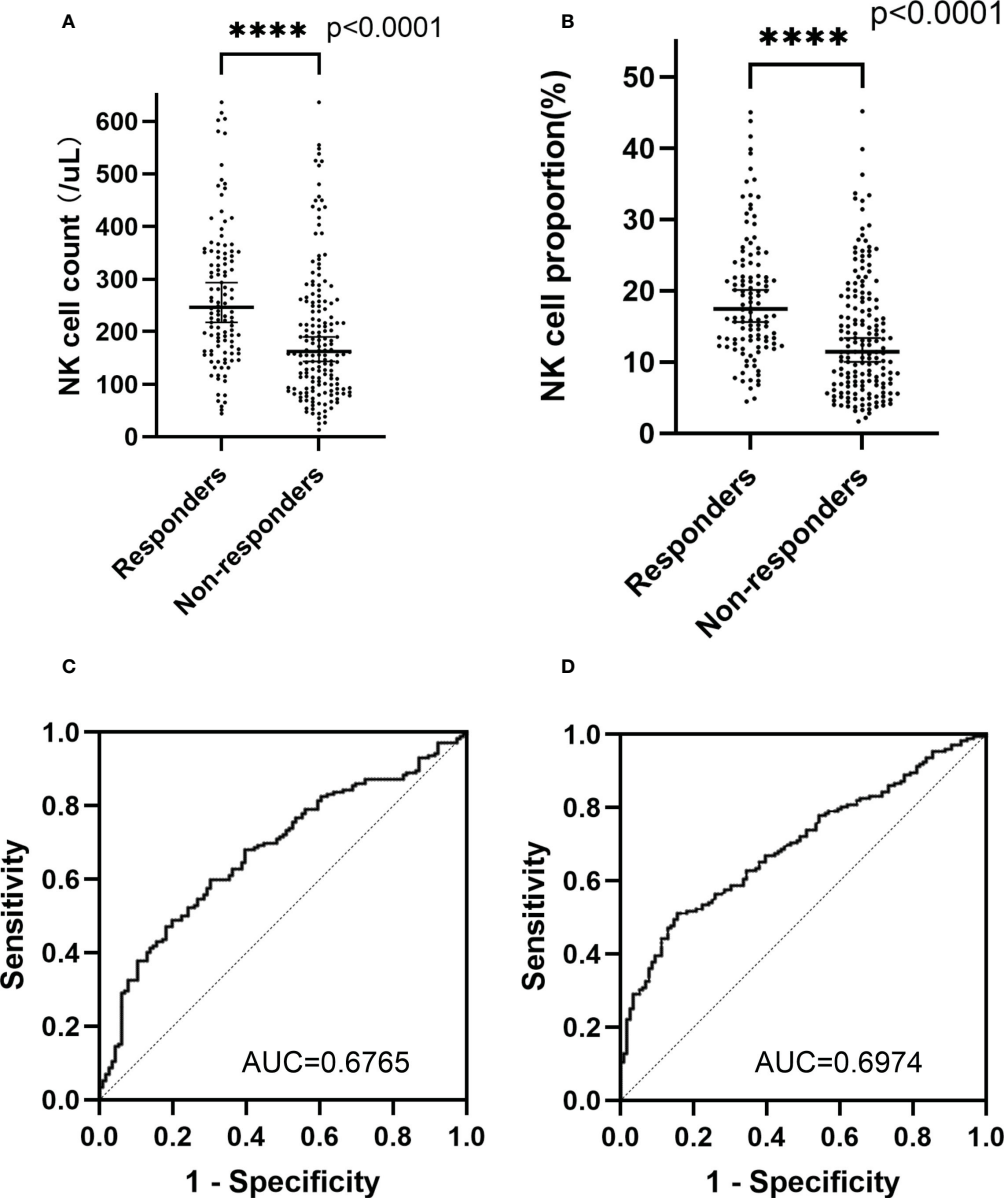
NK cell count and NK cell proportion of different groups. **(A)** NK cell count in the peripheral blood of responders and non-responders **(B)** NK cell proportion in the peripheral blood of responders and non-responders **(C)** ROC curve of NK cell count **(D)** ROC curve of NK cell proportion. The symbol **** means that p value <0.0001.

**Figure 2:**
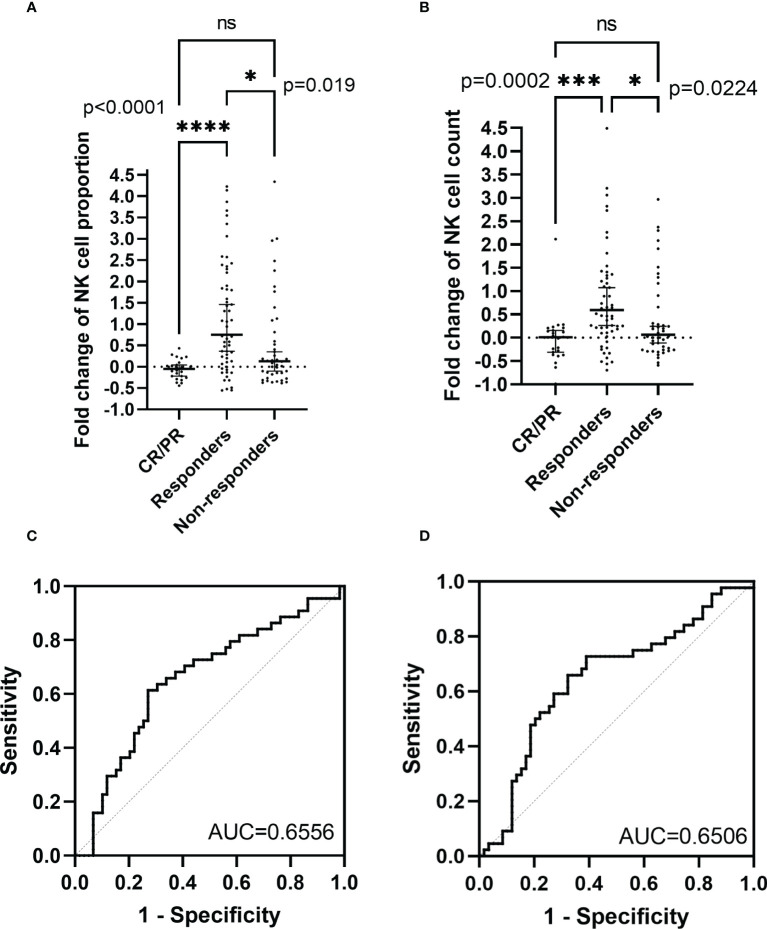
Fold change in NK cell proportion and NK cell count of different groups. **(A)** Fold change in NK cell proportion of responders, non-responders, and patients in complete remission/partial remission **(B)** Fold change in NK cell count of responders, non-responders, and patients in complete remission/partial remission **(C)** ROC curve of fold change in NK cell proportion **(D)** ROC curve of fold change in NK cell count. The symbol **** means that p value <0.0001. The symbol *** means that p value >0.0001 and p value<0.001. The symbol * means that p value >0.01 and p value <0.05. The ns means that p value >0.05.

Responders had a higher median NK cell count (*p*<0.0001; median NK cell count, 246.12 cells/μL vs 162.47 cells/μL) and NK cell proportion (*p*<0.0001; median NK cell proportion, 17.44% vs 11.42%) than non-responders (**Figures 1A, B**). The NK cell count and the proportion of NK cells could partially be a predictor of disease state with an area under curve (AUC) of 0.6765 and 0.6974, respectively (**Figures 1C, D**). For patients who had undergone two consecutive NK cell analyses before and after chemotherapy, 18.90% (24/127) of patients remained in CR/PR; 46.46% (59/127) of patients (responders) achieved CR/PR from a disease state of PD/SD after chemotherapy; and 34.65% (44/127) of patients (non-responders) achieved a disease state of PD after chemotherapy. The median fold change in NK cell proportion was 0.20 (range -0.55–8.88) and the median fold change in NK cell count was 0.22 (range -0.99–10.98). Responders had a higher median fold change in NK cell proportion than non-responders (*p*=0.019; median fold change in NK cell proportion, 0.75 vs 0.13) or patients in PR/CR (*p*<0.0001; median fold change in NK cell proportion, 0.75 vs -0.05). Similarly, responders had a higher median fold change in NK cell count than non-responders (*p*=0.0224; median fold change in NK cell count, 0.59 vs 0.07) or patients in PR/CR (*p*=0.0002; median fold change in NK cell count, 0.59 vs 0.01) (**Figures 2A, B**). The fold change in NK cell proportion and NK cell count could be a predictor of treatment response with an AUC of 0.6556 and 0.6506, respectively (**Figures 2C, D**).

### Characteristics of patients with newly diagnosed PCNSL

Eighty-nine newly diagnosed patients with PCNSL from December 2018 to December 2019 at our institution were identified from the medical records and were selected for the study. Patients with histologically confirmed DLBCL or cytologically confirmed B-cell lymphoma from vitreous and/or CSF aspirates were included. Each included patient had measurable lesions from contrast-enhanced magnetic resonance images. Twelve patients who were lost to follow-up were excluded. The final cohort consisted of 77 patients. All patients received a high-dose methotrexate-based regimen (without rituximab) for induction therapy. The median follow-up was 31 months (95% CI: 28.5 to 32.4). The median PFS was 7 months (95% CI: 3 to 16 months) and the median OS was not reached (95% CI: 25 to not estimable). The clinical data of patients with newly diagnosed PCNSL are summarized in **Table 2**. The median age of the cohort was 61 years (range: 24–79). The median KPS was 50 (range: 10–90). Twenty-two (28.57%) patients had undergone surgical excision before chemotherapy. Thirty-four (44.16%) patients had multifocal lesions (≥2). Fifty-six (72.73%) patients had tumors involving deep structures, including the periventricular regions, basal ganglia, corpus callosum, brainstem, and cerebellum. Twenty-six (35.62%) of the 73 patients had lymphoma cells in the CSF and 10 (13.70%) patients had lymphoma cells in the vitreous fluid. The remaining 4 patients did not undergo lumbar puncture and vitrectomy due to fragility. Forty-two (54.54%) patients had high LDH values (>192 U/L). Multifocal lesions, CSF involvement, and high LDH were predictors of shorter PFS; low KPS, multifocal lesions, and high LDH were predictors of shorter OS. The median proportion of NK cells and the median NK cell count of the cohort were 10.54% (range: 1.68–39.83) and 158.14 cells/μL (range: 13.11–554.89), respectively, which were evenly distributed in patients with different ages, sex, LDH, numbers, and locations of lesions, and CSF involvement or not. Patients with high KPS had a higher NK cell count than their counterparts (*p*=0.0210). Patients who had undergone surgical excision had a slightly higher proportion of NK cells than those who did not receive surgical excision (*p*=0.0414). Patients with vitreous fluid involvement had a higher proportion of NK cells (*p*=0.0162) and a higher NK cell count (*p*=0.0134) than those without vitreous fluid involvement (**Table 3**).

**Table 2:** Clinical data of 77 patients with newly diagnosed PCNSL.

Characteristics	All patients (n=77)	Median PFS (Months, 95%CI)	*p*-value	Median OS (Months, 95%CI)	*p*-value
Age					
Median (range) >61 y ≤61 y	61 (24–79) 36 (46.75%) 41 (53.25%)	5 (3.0–24.5) 10 (2.2–17.5)	0.7597	26 (18–) - (27–)	0.2485
KPS					
Median (range) >50 ≤50	50 (10–90) 38 (49.35%) 39 (51.75%)	5 (2.2–24.8) 7 (3–14)	0.7689	- (–) 25 (14–)	0.0157
Diagnostic method					
Non–surgical excision Surgical excision	55 (71.43%) 22 (28.57%)	10 (3.8–19) 3 (1–)	0.9920	- (25–) - (13.2–)	0.6738
Number of lesions					
Multifocal lesions Solitary lesion	34 (44.16%) 43 (55.84%)	2.3 (1.2–9) 19 (4.5–34.2)	0.0011	19.7 (14–) - (–)	0.0030
Lesion location					
Deep structures Superficial structures	56 (72.73%) 21 (27.27%)	7 (3–16) 6.8 (2.3–)	0.3010	- (23.3–) - (14–)	0.6234
CSF involvement	n=73				
Yes No	26 (35.62%) 47 (64.38%)	3 (2.2–10) 19 (4.5–)	0.0049	- (11–) - (27–)	0.3047
Vitreous fluid involvement	n=73				
Yes No	10 (13.70%) 63 (86.30%)	19 (0.5–) 6.5 (2.5–12.7)	0.5469	27 (4–) - (26–)	0.5388
LDH (U/L)					
Median (range) >192 ≤192	202 (120–441) 42 (54.54%) 35 (45.46%)	4.5 (2.8–10) 19 (2.5–)	0.0166	19.7 (13.2–) - (–)	0.0009

PCNSL, primary central nervous system lymphoma; KPS, Karnofsky performance status; CSF, cerebrospinal fluid; LDH, lactate dehydrogenase.

**Table 3:** Distribution of NK cell proportion and NK cell count in each group.

	NK cell proportion (n=77)(%)	P value	NK cell count (n=77) (cells/μL)	*p* value
Age				
Age>61 y Age ≤ 61 y	11.33 (2.18–39.83) 8.31 (1.68–33.41)	0.0947	148.21 (44.41–554.89) 165.68 (13.11–537.93)	0.6661
Sex				
Male Female	10.02 (1.68–33.41) 10.55 (2.18–39.83)	0.5050	155.42 (13.11–525.36) 163.00 (44.41–554.89)	0.9338
KPS				
KPS>50 KPS ≤ 50	10.58 (3.28–39.83) 9.89 (1.68–32.92)	0.5018	198.74 (35.58–554.89) 143.74 (13.11–480.06)	0.0210
Diagnostic method				
Surgical excision Non–surgical excision	11.91 (3.93–32.92) 7.87 (1.68–39.83)	0.0414	193.08 (44.41–480.06) 154.86 (13.11–554.89)	0.4295
Numbers of lesions				
Multifocal lesions Solitary lesion	9.81 (1.68–33.41) 10.54 (3.15–39.83)	0.9451	148.21 (13.11–457.00) 174.43 (35.58–554.89)	0.1921
Location of the lesion				
Deep structures Superficial structures	9.2 (1.68–39.83) 11.1 (3.15–32.92)	0.9665	155.14 (13.11–554.89) 183.86 (35.58–537.93)	0.2873
CSF involvement				
Yes No	n=73 8.19 (3.28–32.92) 10.54 (1.68–39.83)	0.7725	175.85 (35.58–537.93) 156.73 (13.11–554.89)	0.3562
Vitreous fluid involvement				
Yes No	n=73 15.64 (4.46–39.83) 8.45 (1.68–33.41)	0.0162	286.80 (104.58–554.89) 154.68 (13.11–525.36)	0.0134
LDH (U/L)				
>192 ≤192	10.98 (1.68–25.87) 9.89 (2.18–39.83)	0.5398	157.44 (13.11–449.42) 165.68 (48.24–554.89)	0.6362

KPS, Karnofsky performance status; CSF, cerebrospinal fluid; LDH, lactate dehydrogenase.

### Prognostic value of peripheral NK cell count in newly-diagnosed PCNSL

**Figure 3:**
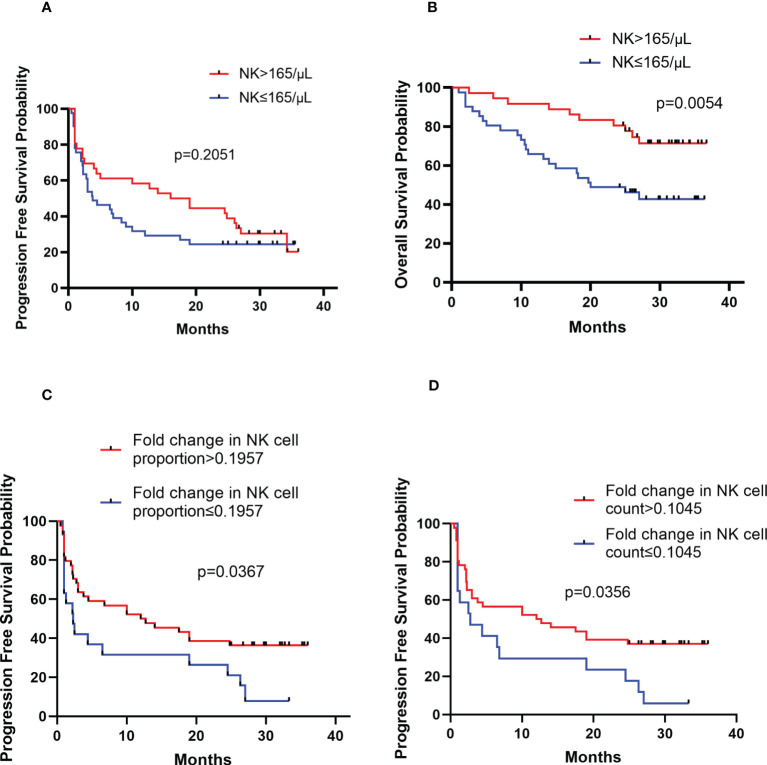
Kaplan-Meier survival curves of patients with newly diagnosed PCNSL **(A)** Kaplan-Meier curves for PFS according to NK cell count **(B)** Kaplan-Meier curves for OS according to NK cell count **(C)** Kaplan-Meier curves for PFS according to the fold change in NK cell proportion **(D)** Kaplan-Meier curves for PFS according to the fold change in NK cell count.

**Figure 4:**
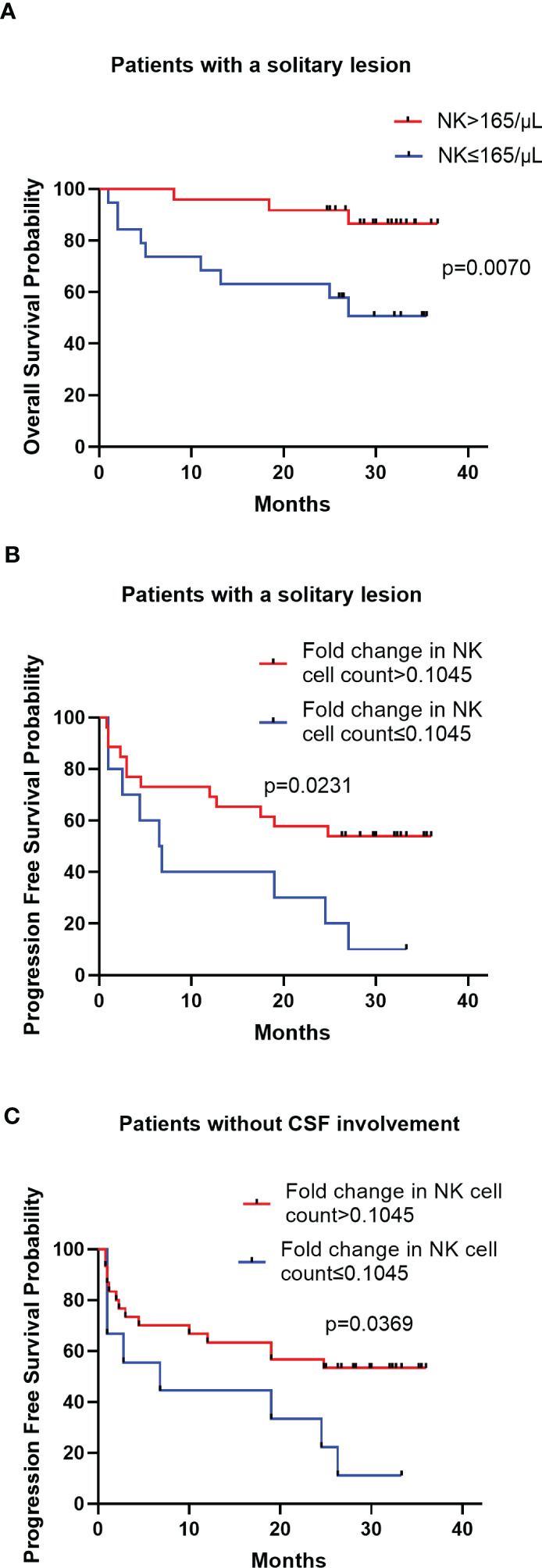
Kaplan-Meier survival curves of patients in stratified analysis **(A)** Kaplan-Meier curves for OS according to NK cell count in patients with a solitary lesion **(B)** Kaplan-Meier curves for PFS according to the fold change in NK cell count in patients with a solitary lesion **(C)** Kaplan-Meier curves for PFS according to the fold change in NK cell count in patients without CSF involvement.

The peripheral NK cell count had no impact on the PFS of PCNSL (*p*=0.2051; **Figure 3A**), but the median OS of patients with high NK cell count (>165 cells/μL) was not reached, longer than those with low NK cell count, who had a median OS of 20 months (*p*=0.0054; **Figure 3B**). The high proportion of peripheral NK cells (>15.9%) had no impact on the PFS (*p*=0.7416) or OS (*p*=0.6320) of the cohort. Among 77 patients with newly diagnosed PCNSL, 63 underwent two consecutive NK cell analyses before and after chemotherapy. The fold change in NK cell count and the proportion of NK cells were calculated for each patient. The results indicated that a high fold change in the proportion of NK cells (>0.1957) had a median PFS of 12.4 months, longer than the counterparts which had a median PFS of 2.3 months (*p*=0.0367; **Figure 3C**). Patients with a high fold change in NK cell count (>0.1045) had a median PFS of 12.4 months, much longer than their counterparts who had a median PFS of 2.8 months (*p*=0.0356) (**Figure 3D**). There was no significant correlation between high fold change in NK cell proportion (*p*=0.5763) or high fold change in NK cell count (*p*=0.7985) with OS. Multivariate Cox regression analysis indicated that CSF involvement (*p*=0.001) and multifocal lesions (*p*=0.003) were independently correlated with shorter PFS (**Table 4**), while low KPS (*p*=0.009) and multifocal lesions (*p*=0.034) were independently associated with shorter OS (**Table 5**). Fold change in NK cell proportion or in NK cell count was not an independent predictor of PFS, and NK cell count was not an independent predictor of OS. In order to further evaluate the prognostic value of NK cells in PCNSL, a stratified analysis was performed to exclude the prognostic impact of KPS, multifocal lesions, and CSF involvement. A high peripheral NK cell count was associated with longer OS in patients with a solitary lesion (*p*=0.0070; **Figure 4A**), but had no impact on OS in patients with low KPS (*p*=0.5684) or high KPS (*p*=0.4508) or multifocal lesions (*p*=0.4763). The fold change in the proportion of NK cells had no impact on PFS in patients with a solitary lesion (*p*=0.0551) or multifocal lesions (*p*=0.192), while a high fold change in the NK cell count was a predictor of longer PFS in patients with a solitary lesion (*p*=0.0231; **Figure 4B**), but not in patients with multifocal lesions (*p*=0.3367). The fold change in the proportion of NK cells was not correlated with the PFS of patients with (*p*=0.5392) or without (*p*=0.1463) CSF involvement. However, high fold change in NK cell count was a predictor of longer PFS in patients without CSF involvement (*p*=0.0369; **Figure 4C**), but not in patients with CSF involvement (*p*=0.8198).

**Table 4:** Multivariate analysis by Cox regression for PFS (n=61).

Factors	*p*-value	Hazard Ratio (95%Cl)
Age LDH Multifocal lesions CSF Fold change of NK cell count Fold change of NK cell proportion	0.490 0.572 0.003 0.001 0.726 0.883	1.009 (0.983–1.037) 1.002 (0.996–1.008) 2.848 (1.434–5.657) 3.118 (1.595–6.093) 1.038 (0.843–1.278) 0.977 (0.715–1.335)

PFS, progression-free survival; LDH, lactate dehydrogenase; CSF, cerebrospinal fluid.

**Table 5:** Multivariate analysis by Cox regression for OS (n=77).

Factors	P-value	Hazard Ratio (95%Cl)
LDH Multifocal lesions NK cell count KPS	0.195 0.034 0.687 0.009	1.003 (0.998–1.008) 2.242 (1.063–4.726) 0.999 (0.996–1.003) 0.972 (0.952–0.993)

LDH, lactate dehydrogenase; Karnofsky performance status.

### *In vitro* cytotoxicity assay and immunohistochemistry assay

**Figure 5:**
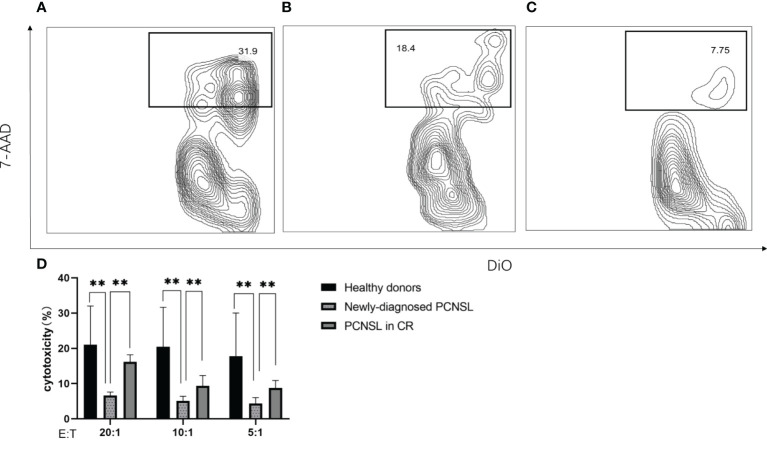
NK cell cytotoxicity measured by flow cytometry **(A)** Representative example of NK cell cytotoxicity from healthy donors **(B)** Representative example of NK cell cytotoxicity from patients with PCNSL in CR **(C)** Representative example of NK cell cytotoxicity from patients with newly-diagnosed PCNSL **(D)** Relative levels of NK cell killing of K562 cells at effector-to-target ratios from 20:1 to 5:1 (n=3 healthy donors, n=3 patients with PCNSL in CR, n=3 patients with newly-diagnosed PCNSL). The symbol ** means that p value >0.001 and p value<0.01.

NK cells from healthy donors demonstrated higher cytotoxic capacity than those from patients with newly-diagnosed PCNSL at effector-to-target ratios from 20:1 to 5:1 (*p*=0.0031); higher NK cell cytotoxicity was observed in patients in CR than in patients with newly-diagnosed PCNSL (*p*=0.0043). No significant difference in NK cell cytotoxicity was observed between healthy donors and patients in CR (*p*=0.13) (**Figure 5**).

In order to analyze the NK cell infiltration in the tumor tissue, immunohistochemistry was performed to evaluate CD56-positive lymphocytes in paraffin-embedded sections from 10 patients with PCNSL. We found that there were few CD56-positive lymphocytes in the TME of PCNSL (**Supplementary Figure 1**).

## Discussion

In this study, we evaluated the prognostic impact of the NK cell proportion and NK cell counts in patients with PCNSL. Previous studies have evaluated the prognostic impact of circulating lymphocytes on PCNSL. A higher neutrophil-lymphocyte ratio has been reported to correlate with worse PFS and OS in PCNSL (26, 27), suggesting that circulating lymphocytes were involved in PCNSL development. CD4+ T cells, other than CD8+ T cells in peripheral blood, may play a more pivotal role in PCNSL (22). However, to our knowledge, the importance of circulating NK cells for PCNSL has not been evaluated. In this study, we found that circulating NK cells, although not in direct contact with tumor cells, demonstrated an impaired cytotoxic capacity compared to patients in CR or healthy donors, suggesting that PCNSL cells have some effect on circulating NK cells. And patients who achieved CR/PR had a higher proportion of NK cells and a higher NK cell count than those who were in SD/PD. A possible explanation was that the reduced tumor volume from chemotherapy alleviated the suppression of NK cells, leading to NK cell expansion. A similar pattern was revealed in ovarian cancer, in which NK cell infiltration and T cell expansion were detected after neoadjuvant chemotherapy (28). Immune suppressive factors in the tumor microenvironment, such as hypoxic exosomes and cytokines, resulted in NK cell dysfunction and immune escape (29). Reduction in NK cell proliferation was also observed in patients with gastric cancer (30) and with DLBCL (31). Tumor cells may interrupt NK cell development in the bone marrow (32). If the tumor bulk is eliminated, NK cell development will be restored, which was consistent with the expansion of NK cells observed only in those patients achieving disease control (from SD/PD to CR/PR). No significant elevation of the proportion/count of NK cells was observed in patients in CR/PR or in those who did not respond to chemotherapy. It could also be inferred that the elevated proportion or number of NK cells was not due to the agents used for treatment. Patients receiving surgical excision had a higher proportion of NK cells than their counterparts without surgical excision in this study. Because all NK cell analyses were performed after surgery when the tumor bulk had been resected, the elevation of NK cell proportion may be the result of disinhibition. Another explanation was that the increase in NK cell proportion/count contributed to disease control. This could also be inferred from our findings showing that a larger expansion of the proportion/count of NK cells after chemotherapy was correlated with a longer PFS. Although multivariate analysis indicated that the elevated NK cell count was not an independent prognostic factor for PFS, the subsequent stratified analysis suggested that it had an impact on the prognosis of patients with a solitary lesion or in those without CSF involvement. For patients with multifocal lesions or CSF involvement, increasing the NK cell count was not sufficient to control disease progression.

A lower NK cell count has been correlated with shorter PFS but not OS in patients with DLBCL, in part because all patients in the cohort had received immunochemotherapy (33). In this study, NK cell count had no effect on the PFS of patients with PCNSL, perhaps because most PCNSL patients received high-dose methotrexate-based treatment and few patients received rituximab. Patients without immunotherapy may not benefit from the antibody-dependent cell-mediated cytotoxicity (ADCC) effect exerted by NK cells. Another study indicated that higher activated NK cells in the TME of DLBCL were associated with a poor outcome (34). The role of NK cells in DLBCL is controversial, partly because NK cells in the peripheral blood and tumor tissues may belong to different subpopulations. Unlike DLBCL, peripheral circulating NK cells play a different role in PCNSL. Four types of immune cells-T cells, B cells, macrophages, and dendritic cells have been identified in PCNSL tumor tissues by single-cell sequencing (35). Consistent with the aforementioned study, immunohistochemical analysis in this study suggested that there were few CD56-positive NK cells in the TME of PCNSL. There is no evidence supporting the presence of NK cells in the PCNSL microenvironment, suggesting that PCNSL has a unique microenvironment that differs from other types of central nervous system neoplasms such as glioma (36), of which NK cells are an important component of the TME. In this study, a lower NK cell count was correlated with a shorter OS in PCNSL, especially in patients with a solitary lesion, suggesting that circulating NK cells were involved in PCNSL disease control. The presence of the blood-brain barrier may make it difficult for NK cells to reach the central nervous system for tumor immunosurveillance in PCNSL. However, the disruption of the blood-brain barrier by chemotherapy agents allows circulating NK cells to enter the central nervous system (37), and thus, circulating NK cells are able to intervene in PCNSL development. From another perspective, NK cells in the peripheral blood are less affected by central nervous system neoplasms and can be used as a potential weapon against central nervous system lymphoma.

Patients with vitreous fluid involvement had a higher proportion and number of NK cells in this study. A previous study indicated that vitreous samples from intraocular lymphoma had higher cellularity than non-lymphomatous samples (38). Whether the involvement of vitreous fluids can trigger immune cell activation and expansion needs to be elucidated.

Our study had several limitations. This was a retrospective cohort study, and selection bias could not be avoided. The sample size in this study was not sufficient to perform validation analysis. Regarding the analysis of NK cells, we used a 6-color TBNK Reagent, in which NK cells are identified as CD3-CD56+ and/or CD16+ cell populations. The assay could not distinguish the CD56dim CD16+ and CD56bright CD16- subsets, thus it was impossible to distinguish which subsets expanded after the tumor bulk was removed. Although the proportion of NK cells was evaluated in this study, the possible interaction between NK cells and other circulating immune cells was not analyzed due to the limited sample size.

Our study indicated that NK cells in the peripheral blood had some impact on the outcome of PCNSL. Patients who were in CR/PR had a higher NK cell count and NK cell proportion than those in SD/PD. NK cell count and NK cell proportion increased more significantly in patients who had achieved CR/PR after chemotherapy than in those who did not respond to chemotherapy. For newly diagnosed PCNSL, a high NK cell count appeared to have a longer median OS than those with a low NK cell count, especially in patients with a solitary lesion. A higher elevation of NK cell count after chemotherapy was a predictor of a longer PFS, especially in patients with a solitary lesion or patients without CSF involvement.

## Data availability statement

The raw data supporting the conclusions of this article will be made available by the authors, without undue reservation.

## Ethics statement

The studies involving human participants were reviewed and approved by the Ethics Committee of Huashan Hospital, Fudan University. The patients/participants provided their written informed consent to participate in this study.

## Author contributions

Material preparation was performed by ZL and JM. Data collection was performed by ZL, JM, YM, and QL. Data analysis was performed by ZL, HK, and MZ. The study design was performed by ZL, BC, and RX. All authors contributed to the article and approved the submitted version.

## Supplementary material

Supplementary Figure 1
**(A–C)** Representative images of immunohistochemical staining for CD56 expression in tumor tissues of PCNSL
