# Supplementary figures and images for: Prognostic impact of peripheral natural killer cells in primary central nervous system lymphoma
Source: Front Immunol. 2023 Jun 22;14:1191033. doi: 10.3389/fimmu.2023.1191033 (PMC10326164; doi:10.3389/fimmu.2023.1191033)

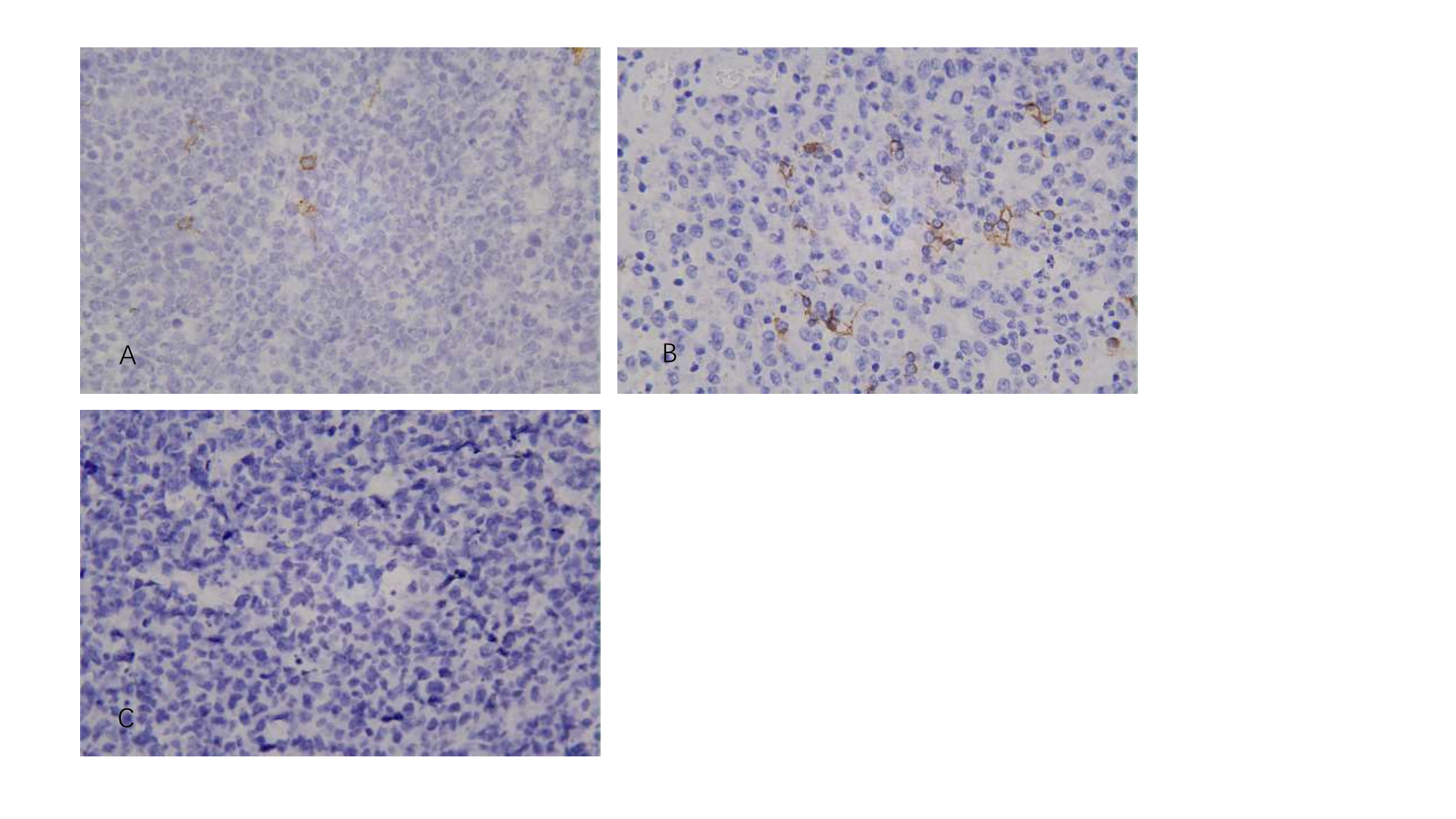

Supplement: Supplementary Figure 1 — (A–C) Representative images of immunohistochemical staining for CD56 expression in tumor tissues of PCNSL [file Image_1.tif]
